# Management of Coccidioidal Meningitis Outside the Endemic Region

**DOI:** 10.7759/cureus.105643

**Published:** 2026-03-22

**Authors:** Alexander D Smith, Alexa R Lauinger, Mahima Goel, Prateek Dullur, Logan Burrington, Anant Naik, Amir Khan, Suguna Pappu

**Affiliations:** 1 Computer Science, Carle Illinois College of Medicine, University of Illinois Urbana-Champaign, Urbana, USA; 2 Medicine, Carle Illinois College of Medicine, Urbana, USA; 3 Neurosurgery, University of Minnesota, Minneapolis, USA; 4 Infectious Diseases, Carle Foundation Hospital, Urbana, USA; 5 Neurosurgery, Carle Foundation Hospital, Urbana, USA

**Keywords:** case report, central nervous system, coccidioidomycosis, endemic infection, leptomeningeal enhancement

## Abstract

Leptomeningeal enhancement (LE) is an MRI neuroimaging characteristic associated with infection, malignancy, or cerebrovascular pathologies of the subarachnoid space. LE secondary to infection is most commonly a sign of meningeal tuberculosis. Fungal infections have a heightened presence in certain endemic regions in North America. We present the case of a 44-year-old male patient from Illinois with persistent headaches, hydrocephalus, and LE on MRI. He was empirically treated for tuberculosis, then blastomycosis, but later was found to have disseminated *Coccidioides immitis* infection via lymph node biopsy. Neurosurgical intervention with external ventricular drainage, then ventriculoperitoneal shunting, was required for hydrocephalus. The patient was treated with intravenous liposomal amphotericin B for suspected blastomycosis infection, then transitioned to oral fluconazole, resulting in resolution of symptoms and no neurological deficits at follow-up. This case is among the few reported instances of coccidioidal meningitis diagnosed outside endemic regions. Neurosurgeons and clinicians should maintain a broad differential for LE on MRI and not exclude endemic fungal infections based solely on geographic history. Prompt initiation of appropriate antifungal therapy is key to improving outcomes in coccidioidal meningitis. This case highlights the need for heightened clinical suspicion and aggressive management of meningitis with LE.

## Introduction

Leptomeningeal enhancement (LE) refers to an abnormal contrast-enhanced visualization of the leptomeninges (arachnoid and pia mater) on MRI [1]. This finding suggests destruction of the blood-brain barrier and infiltration of the subarachnoid space by infectious or inflammatory pathogens. The most common causes of LE are infectious etiologies, including bacterial or viral meningitis in immunocompetent patients and fungal, viral, or tuberculous meningitis in immunocompromised patients. Neoplastic causes, including leptomeningeal carcinomatosis, are also common, as well as CNS lymphoma and autoimmune conditions like neurosarcoidosis or multiple sclerosis. Rarely, irregular vascularity, as seen in subarachnoid hemorrhage or cerebral venous thrombosis, can elicit LE.

Due to the rarity of patients presenting with LE, there is limited literature detailing concurrent underlying etiologies. In addition, few cases have reported on CNS involvement of opportunistic infections in immunocompetent patients. Specifically, the acquisition and CNS dissemination of fungal infections, including coccidioidomycosis and blastomycosis, in immunocompetent patients are very rare, with some reports suggesting an incidence of less than 1% [2,3]. We present a case of an immunocompetent patient with LE and hydrocephalus who was treated for several infectious diseases, including tuberculosis and blastomycosis, before a definitive diagnosis of disseminated coccidioidomycosis with intracranial manifestation was made and appropriate treatment was delivered.

Coccidioidomycosis is endemic to the Southwestern United States, especially the San Joaquin Valley, but cases outside the endemic region are very rare [4]. Coccidioidomycosis typically manifests as a pulmonary disease, but occasionally spreads lymphatically or hematologically to other organ systems, including infiltration into the central nervous system [2]. Only three known cases of coccidioidal meningitis (CM) have been reported outside endemic regions, with relatively complicated management, as in the present case report [5-7]. As global travel has become increasingly common in modern society, we argue that clinicians must maintain heightened awareness of non-endemic diseases, even in regions where they are not normally encountered, and should routinely obtain a multi-year travel history to ensure that definitive medical management is provided.

## Case presentation

**Figure 1:**
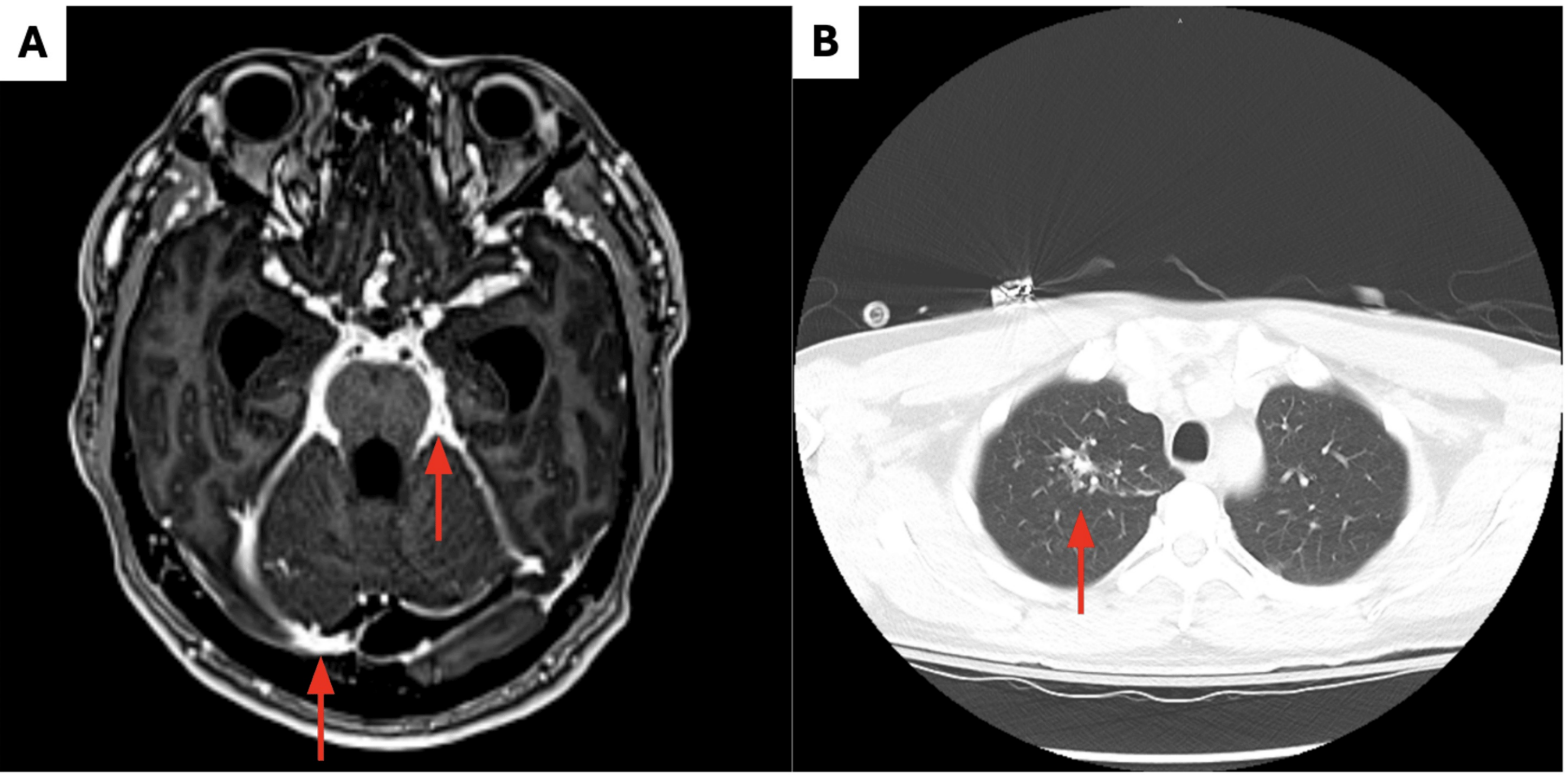
Patient imaging studies. (A) Axial T1 contrast MRI indicating leptomeningeal enhancement and subdural abscesses (arrows). B) Chest CT with right superior lobar nodular opacities in a tree-in-bud pattern (arrow).

A 44-year-old Spanish-speaking male who immigrated to Illinois from Mexico two years prior presented to the emergency department in July 2024 with two weeks of persistent nausea, vomiting, and decreased oral intake with no associated fever, abdominal pain, neck stiffness, hemoptysis, or interaction with recent sick contacts. Despite discharge with famotidine for a suspected diagnosis of gastritis, he returned to the hospital three weeks later with no improvement in his nausea or vomiting, with additional new-onset bilateral periorbital headaches. On CT imaging, hydrocephalus was noted, and MRI uncovered extensive LE in the posterior fossa, cervical canal, and suprasellar cisterns extending to the sylvian fissures bilaterally (Figure 1A). A chest X-ray at the time showed no acute pulmonary process. The patient was admitted, and an external ventricular drain (EVD) was placed at the bedside in the anterior horn of the right ventricle, expressing pink-tinged cerebrospinal fluid (CSF) at 10 cm H₂O.

CSF analysis showed a lymphocytic pleocytosis with low glucose and slightly elevated protein. The patient denied a prior history of tuberculosis or exposure to others with tuberculosis, but did endorse growing up on farmland with constant exposure to chickens, pigs, and other farm animals. The patient had no history of diabetes, HIV, or long-term corticosteroid use and was not a prior organ recipient. There were no other prior concerns for immune suppression. During workup, ANA, CRP/ESR, blood culture, QuantiFERON, RPR, and HIV serology were all negative. Chest CT demonstrated multiple nodular opacities in a tree-in-bud pattern in the superior right upper lobe with bronchial wall thickening (Figure 1B). Additionally, enlarged superior mediastinal and paratracheal lymph nodes were identified, suggestive of an atypical infectious process or infective bronchiolitis. Pulmonary tuberculosis was the highest on the differential diagnosis, so airborne isolation measures were established. Empiric treatment with isoniazid, rifampicin, pyrazinamide, and ethambutol was administered.

An endobronchial ultrasound-guided lymph node biopsy with lavage was performed shortly after to confirm the diagnosis. Initial pathology from the right paratracheal lymph node showed abundant broad-based budding yeasts, raising concern for possible blastomycosis, and elevated *Blastomyces* and *Coccidioides* antibodies were detected at that time. An acid-fast bacilli (AFB) smear from the bronchoalveolar lavage was negative, as was the mycobacterium PCR. The remaining sample was tested and sent for culture. Tuberculosis treatment and isolation protocols were suspended, and intravenous liposomal amphotericin B was administered instead for a preliminary diagnosis of pulmonary blastomycosis.

Approximately two weeks after admission, there was evidence of reduced drain function, and per protocol, the first EVD was replaced. For additional data during the revision of the frontal, the burr hole was extended, and a dural biopsy was performed to obtain additional tissue for diagnosis. The dural specimen was noted to be inflamed and boggy but was negative for any growth. Aerobic and anaerobic CSF cultures were also negative. Disseminated blastomycosis remained the leading differential.

**Figure 2:**
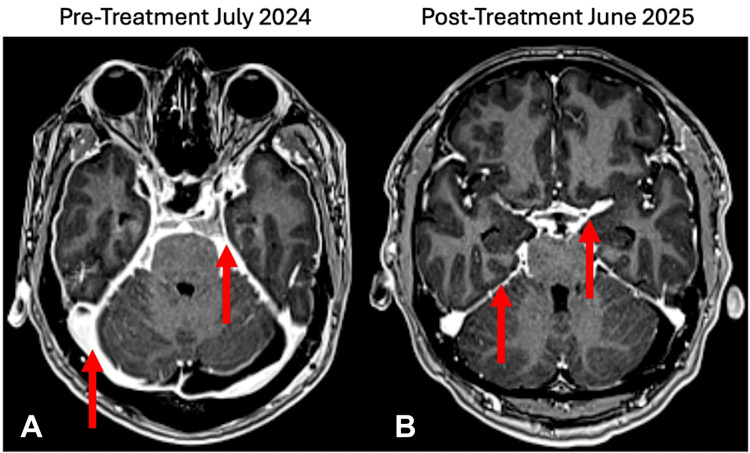
10-month follow-up cranial imaging study. (A) Pre-treatment in July 2024. (B) Post-treatment in June 2025. Mild reduction in leptomeningeal enhancement of the basal cisterns and reduction in diffuse pachymeningeal enhancement are noted with the arrows.

After 10 days of treatment with amphotericin B, fungal cultures from the right paratracheal lymph node fine-needle aspiration grew *Coccidioides immitis*, and *Coccidioides* serology came back positive for both immunodiffusion and complement fixation. A definitive diagnosis of CM was made, and the patient remained on amphotericin B for the remainder of his admission. A ventriculoperitoneal shunt was placed, and after six weeks, amphotericin B was transitioned to oral fluconazole for 12 months. Following shunt placement and antifungal treatment, the patient gradually became asymptomatic and was discharged on famotidine and ondansetron for nausea management, displaying no other lingering symptoms. As of the most recent visit in June 2025, the patient reports no functional neurological deficits and demonstrates complete resolution of symptoms after 10 months of oral fluconazole treatment. A repeat MRI indicates a reduction in LE and diffuse pachymeningeal enhancement (Figure 2).

**Figure 3:**
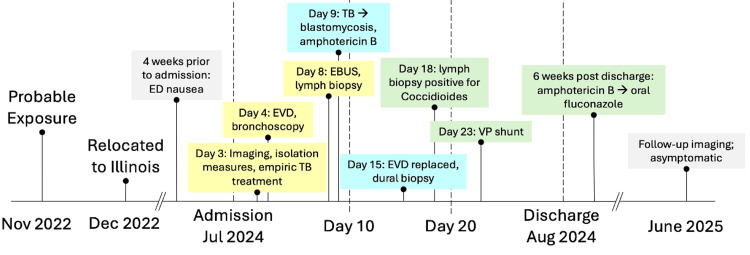
Timeline of patient disease state and management. ED: emergency department, EVD: external ventricular drain, TB: tuberculosis, EBUS: endobronchial ultrasound, VP: ventriculoperitoneal

During an extensive inpatient stay, the patient was initially diagnosed with and empirically treated for tuberculosis, as well as being placed on respiratory precautions. After management of elevated intracranial pressure and identification of a lymph node inclusion, initial pathology identified broad-based budding yeasts, increasing suspicion for disseminated Blastomycosis infection. Both blastomycosis and coccidioidomycosis antibodies were elevated. Subsequently, tuberculosis treatment was replaced with blastomycosis treatment. At EVD replacement, a dural biopsy was taken, showing no growth on culture. Cultures from a paratracheal lymph node biopsy confirmed an unexpected yet definitive diagnosis of disseminated coccidioidomycosis, and appropriate treatment was administered approximately one month after initial admission. The patient's disease and treatment route are shown in Figure 3.

Written informed consent for the use of imaging and history from this case was obtained from the patient on August 26, 2024.

## Discussion

This case was uniquely challenging for several reasons: a rare case of delayed-onset coccidioidomycosis causing LE, the geographic location in which the diagnosis was made, and the management of this infection due to the unexpected diagnosis and evaluation indicative of potential tuberculosis or blastomycosis during the treatment route.

Coccidioidomycosis is an endemic fungal infection that most commonly causes pneumonia in immunocompromised individuals and is found in the Southwest United States, Northern Mexico, and Central and South America [8]. Notably, this case occurred in rural Illinois, well outside the known endemic regions, which initially raised questions about how the disease was acquired. The etiology became clearer, however, when it was discovered that the patient had entered the US in November 2022, traveling through Arizona before moving to the Midwest in December 2022. CM is a rare manifestation of the fungus that can cause headaches, hydrocephalus, and cerebrovascular complications [9]. There are two species of *Coccidioides*, with *C. immitis* most commonly located in California and Washington state and *C. posadasii* more commonly in Central and South America. Although there are no distinct phenotypic differences between these species, there have been several case reports of CM from *C. posadasii* in immunocompetent patients [5,10]. This case represents a rare CM infection in an immunocompetent patient caused by *C. immitis*. Limited work has been published on the variation between these species, and there are currently no recommended treatment variations by species. Future research should explore how variations may impact the likelihood for dissemination or treatment efficiency.

The diagnosis of CM is most often made via lumbar puncture and CSF analysis for *Coccidioides* antigen [9]. Despite this, diagnosis is often elusive and results from various tests, including CSF titers, dural biopsy, repeat lumbar puncture [11], and peripheral studies such as lymph node biopsy and serum antibodies, as was necessary in this case. Current workup recommendations for CM with hydrocephalus suggest oral fluconazole for medical management and referral to neurosurgery for hydrocephalus management with a shunt [2].

Outcomes following CM remain poor with high rates of rehospitalizations (59%), shunt revisions (52.5%), and complications leading to death (21.8%) [12]. Management of this case was impacted by an atypical presentation and several diagnoses that were challenging to differentiate between: CM, blastomycotic meningitis, and meningeal tuberculosis. Each endemic dimorphic mycosis commonly found in the United States, including *Histoplasma*, *Coccidioides*, and *Blastomyces*, has recognized geographic regions in which it is historically found. In central Illinois, *Histoplasma* is more prevalent than the other two DMs [3,13], making infection with coccidioidomycosis or blastomycosis less likely. In addition, meningeal tuberculosis is more common than meningitis from any of the mycoses, and it most commonly affects the leptomeninges and presents with LE [14]. Opposed to treatment with fluconazole for CM, *Blastomyces* meningitis is treated with amphotericin B or itraconazole, and meningeal tuberculosis is treated with high-dose rifampicin added to the standard anti-tuberculosis regimen. Prior research and the patient history complicated this case and its management, informing the decision to take a dural biopsy during EVD replacement. The different management for the corresponding infections caused a delay in treatment of the primary fungal meningitis and an increased risk of drug toxicity from the anti-tuberculosis regimen.

## Conclusions

Due to the complexity of coccidioidomycosis diagnosis, a critical aspect of patient care, especially in patients who require early intervention for life-saving treatment, is appropriate screening for potential diseases. *Blastomyces*, *Histoplasma*, *Coccidioides*, and tuberculosis of the meninges often cause LE, and due to the variation in presentation within each disease, none can be ruled out in the context of LE and intracranial pathology alone. Therefore, we urge others to consider endemic diseases and travel history in their differential diagnosis for patients presenting similarly. In cases of LE, early screening is essential and should include antibody testing and a thorough search for primary lesions, especially in the lungs, even when not visible on chest X-ray. When feasible, diagnostic efforts should also include lesion biopsy, particularly from the surgical site.

Additionally, based on the observed number of CM caused by *C. immitis* compared to *C. posadasii*, further investigation and review of the clinical presentation differences between the species of *Coccidioides* is warranted. It may be possible that *C. immitis* poses a greater risk of CM compared to *C. posadasii*. Common imaging characteristics can be considered, but further investigation is required for nonspecific signs, such as LE. In difficult cases with hydrocephalus present, tissue biopsies can be considered to verify a diagnosis and finalize the treatment plan. Regarding intracranial coccidioidomycosis cases, particularly those diagnosed outside of the North American Southwest, it is relevant to determine patient travel history, even years before the causal event, and to screen for multiple fungal infections, even if others are suspected due to geography. Misdiagnosis of fungal infections can delay curative treatment, potentially leading to serious implications toward patient health and well-being.
